# False Negative Hepatobiliary Iminodiacetic Acid (HIDA) Scan in a Case of Gall Bladder Perforation

**DOI:** 10.7759/cureus.14247

**Published:** 2021-04-01

**Authors:** Ramakanth Pata, Shristi Lamichhane, Nirajan Regmi, Abolfazl Ahmady, Roudabeh Kiani

**Affiliations:** 1 Pulmonary Medicine, Interfaith Medical Center, Brooklyn, USA; 2 Internal Medicine, Interfaith Medical Center, Brooklyn, USA; 3 Internal Medicine, The Wright Center for Graduate Medical Education, Scranton, USA; 4 Pulmonary, Interfaith Medical Center, Brooklyn, USA

**Keywords:** gall bladder perforation, perforation, gall bladder, diagnosis, hida

## Abstract

Gall bladder perforation (GBP) is a rare and life-threatening complication of acute cholecystitis that requires immediate intervention. The diagnosis itself poses a diagnostic challenge, if the patient presents after the perforation of the gall bladder, especially if the initial imaging techniques such as ultrasonogram (US), computed tomography (CT) scan, hepatobiliary iminodiacetic acid (HIDA) scan and magnetic resonance cholangiopancreatography (MRCP) are inconclusive. Subtle clues such as free fluid around gall bladder and contracted gall bladder should warrant the clinician as these might be the only clues suggestive of gall bladder perforation. Here we describe a case of GBP successfully diagnosed by peritoneal drainage and analysis and subsequently managed by endoscopic retrograde cholangiopancreatography (ERCP) and open cholecystectomy.

## Introduction

Gall bladder perforation (GBP) is a rare and life-threatening complication of acute cholecystitis that requires immediate intervention [1]. The overall median mortality rate is 10.8% [2]. Early diagnosis with medical and surgical management might lower the morbidity and mortality associated with the condition. However, the diagnosis of gall bladder perforation is often a challenge when imaging techniques such as ultrasonogram (US), computed tomography (CT) scan, hepatobiliary iminodiacetic acid (HIDA) scan and magnetic resonance cholangiopancreatography (MRCP) are inconclusive [3,4]. Moreover, it becomes even difficult when the patient has presented after perforation and with nonspecific signs and symptoms. In such cases, subtle findings such as free fluid around gall bladder and contracted gall bladder should warrant the clinician as these might be the only clues suggestive of gall bladder perforation.

Here we describe a case of GBP successfully diagnosed by peritoneal fluid drainage and analysis and subsequently managed by endoscopic retrograde cholangiopancreatography (ERCP) and open cholecystectomy.

## Case presentation

**Figure 1:**
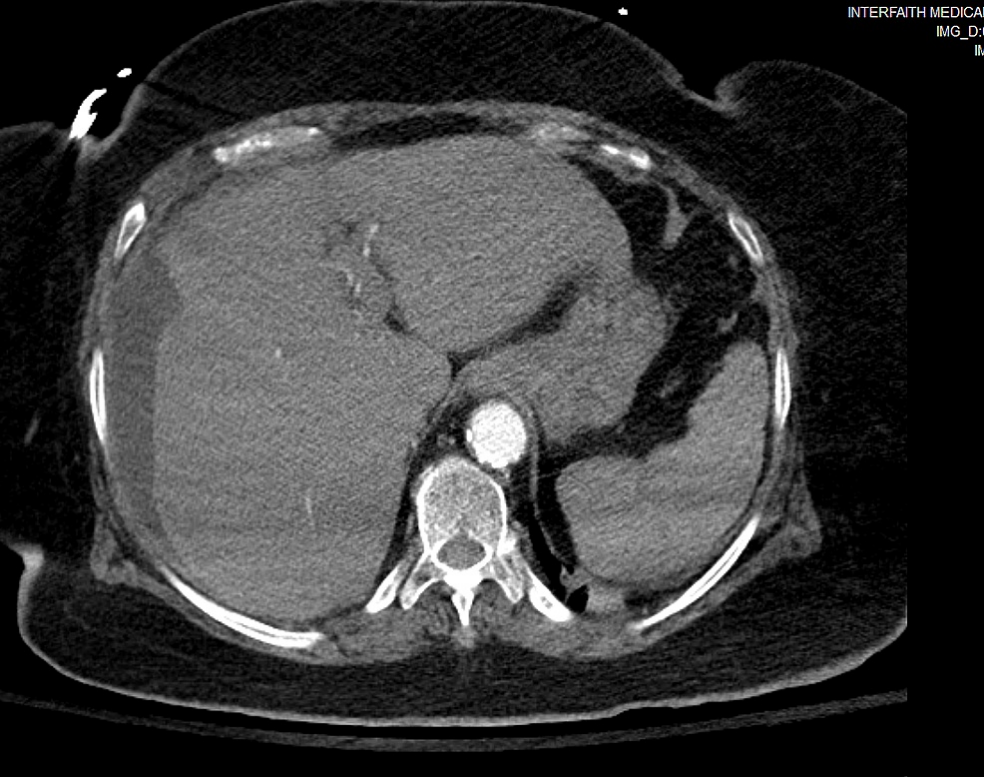
CT abdomen showing peri-hepatic fluid

**Figure 2:**
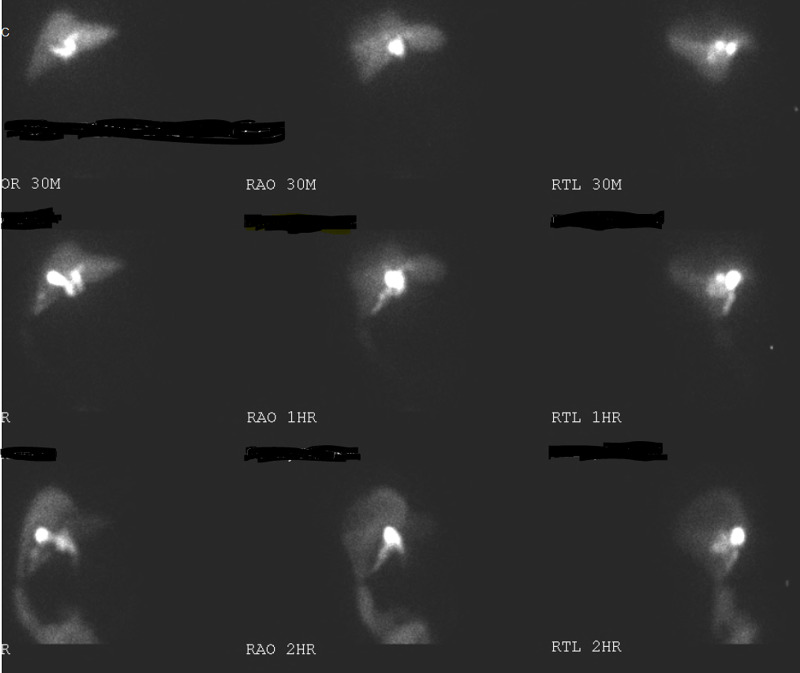
Hepatobiliary iminodiacetic acid (HIDA) scan showing no scintigraphic evidence of biliary obstruction, delayed visualization of gall bladder and bowel may represent chronic cholecystitis, patent common bile and cystic duct, and tracer accumulating along the right abdomen

**Figure 3:**
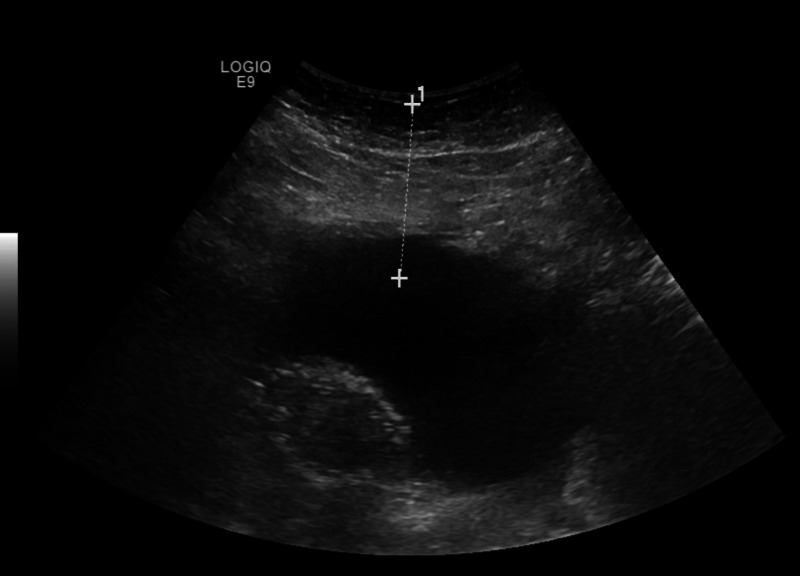
Ultrasonogram (US) abdomen showing free fluid collection in right lower quadrant which was later confirmed to be biliary

An 80-year-old Hispanic lady with past medical history of diabetes mellitus type 2 and hypertension presented to the Intensive Care Unit (ICU) with fever, hypotension and leukocytosis. Her vitals on presentation were: Pulse- 88/min, Temperature- 102.5F, BP- 86/49 mmHg, SpO2 87% on room air. On evaluation, she was found to have severe sepsis, acute respiratory distress and a high sequential organ failure assessment score (SOFA score). She immediately developed pulseless ventricular tachycardia with return of spontaneous circulation within 5 minutes. She was then intubated, started on intravenous fluids as per SEPSIS protocol, vasopressor (Norepinephrine) to maintain target mean arterial pressure (MAP) of 65 and broad-spectrum antibiotics. Chest X-ray was negative for acute pathology. Peripheral blood culture and urine culture did not show any growth. No focus of infection was found. Lab was unremarkable except for elevated blood urea nitrogen (BUN) and creatinine (Table 1). Based on her history of right upper abdominal pain, possibility of cholecystitis was considered. Initial US abdomen showed multiple gallstones, but did not meet sonographic criteria for acute cholecystitis. Initial CT scan of abdomen (Figure 1) revealed fluid around liver but no other acute pathology. Then, HIDA scan (Figure 2) was done in hope of finding a clue which also reported no evidence of acute cholecystitis. A multidisciplinary decision was made to perform bedside sonogram of abdomen (Figure 3) which demonstrated a small to moderate volume of free fluid adjacent to the inferior right hepatic lobe, as well as a larger fluid collection in the right lower quadrant. Ultrasound-guided peritoneal fluid drainage from the right lower quadrant was performed, a pigtail was placed and fluid was sent for analysis. The fluid was confirmed to be biliary as evidenced by the presence of bilirubin, cholesterol, elevated alkaline phosphate and alkaline pH. She then, showed tremendous improvement over the next 12 hours with improvement in SOFA score and hemodynamic status. A repeat abdominal CT scan showed a shrunken gallbladder consistent with the suspected gallbladder perforation, and a 5.8-mm stone in the distal common bile duct (CBD). On day 6 of presentation, an ERCP was performed which removed three calculi from the CBD. Open cholecystectomy was performed on day 7. The gall bladder was found to have areas of hemorrhage and focal greenish-gray exudate adjacent to an irregular defect of the wall within the distal 3rd of the gallbladder. The mucosa was gray-pink finely granular with focal areas of hemorrhage and mucosa raised yellow-green areas and also necrotic areas around the defect. Following the procedure, the patient became conscious, responded to commands and was off the vasopressors and extubated on day 10. The patient was then successfully discharged to a premorbid level.

**Table 1:** Lab values WBC: White blood cell; BUN: Blood urea nitrogen; AST: Aspartate transaminase; ALT: Alanine transaminase; ALP: Alkaline phosphatase; ABG: Arterial blood gas; PCR: Polymerase chain reaction; RBC: Red blood cell; LDH: Lactate dehydrogenase.

Parameters	Reference range	
Hemoglobin	11-15 (g/dl)	11.7
Hematocrit	35-46 (%)	34.6
WBC	4.5-11 (10^3^/uL)	17.3
Platelets	130-400 (10^3^/uL)	259
Lymphocytes	22-48 (%)	4.3
Neutrophils	40-70 (%)	86.3
Sodium	136-145 (mmol/L)	147
Potassium	3.5-5.1 (mmol/L)	3.5
BUN	9.8-20.1 (mg/dl)	39.3
Creatinine	0.57-1.11 (mg/dl)	1.24
Glucose	70-105 (mg/dl)	247
HbA1c	4.8-5.6 (%)	9.1
Lipase	8-78 (U/L)	21
Serum bilirubin	0.2-1.2 (mg/dl)	0.8
AST	5-34 (U/L)	12
ALT	10-55 (U/L)	<10
ALP	40-150 (U/L)	64
Calcium	8.8-10 (mg/dl)	7.7
Albumin	3.2-4.6 (g/dl)	2.9
Phosphorus	2.3-4.7 (mg/dl)	2.7
Magnesium	1.6-2.6 (mg/dl)	2.8
Troponin I	0.00-0.03 (ng/ml)	<0.03
D-dimer	0-500 (ng/ml)	3122
B-natriuretic peptide	10-100 (pg/ml)	229.66
Lactic Acid	0.5-1.9 (mmol/L)	1.9
ABG		PH 7.353, PCO2 49.1, PO2 82.9, on Ventilator FiO2 80%
Covid-19	PCR	Not detected
Sputum Culture		Hemophilus parainfluenza positive
Peripheral Blood Culture		No growth
Urine culture		No growth
Peritoneal fluid culture		No growth
Peritoneal fluid	Color	Brown
	Appearance	Turbid
	PH	7.7
	WBC (cell/uL)	750
	RBC (cell/uL)	1750
	Neutrophils (%)	97
	Lymphocytes (%)	1
	Monocytes (%)	2
	Glucose (mg/dl)	224
	Protein (g/dl)	2.4
	Albumin (g/dl)	1.2
	LDH (IU/L)	1265
	Amylase (U/L)	28
	Triglyceride (mg/dl)	45

## Discussion

The presumptive diagnosis of gall bladder perforation is a challenge when patient presents with features of sepsis and imaging modalities such as ultrasonography, CT scan, MRCP are also inconclusive [4]. The HIDA scan which is more sensitive to diagnose gall bladder perforation is also not useful in case of septic patients as in our case [5]. Our case presented with features of sepsis from gallbladder perforation. Based on risk factors, remote history of abdominal pain and elevated alkaline phosphatase, acute cholecystitis was suspected and imaging were obtained. HIDA scan was done to rule out cholecystitis, which was inconclusive. Ultrasound of the abdomen also did not report evidence of acute cholecystitis, similar inconclusive finding was described by Khan et al. [6]. The HIDA scan not detecting perforation in our case could possibly be due to dye leaking into the abdominal cavity from perforation of gall bladder, which is likely a false negative test. It must be remembered that in the context of worsening SOFA score, a delayed (1 hour) tracer activity noted in the gall bladder with seemingly suspected unusual intrahepatic location may be a clue for gall bladder perforation despite patent cystic duct.

Ultrasound is the best screening modality for biliary diseases [7]. Usual ultrasonographic signs of acute cholecystitis like GB wall thickening and distension, positive sonographic Murphy sign and pericholecystic free fluid may also be evident in cases with gall bladder perforations [8]. The only reliable sign of gallbladder perforation on ultrasound is hole sign with visualization of gall bladder wall defect which can be detected in 70% of patients using a high resolution ultrasound scanner device [8,9].

CT abdomen may offer complementary information and can reveal more accurate signs of free intraperitoneal fluid, pericholecystic fluid, abscess, a distended gallbladder with wall thickening and the defect on the wall due to perforation [7,9]. Gallstones may or may not be seen on CT scan. Sensitivity of CT in the detection of GBP and biliary calculi is 88 and 89%, respectively [10]. HIDA scan may be performed when US and CT are equivocal for cholecystitis or when there is suspicion for bile leak [7]. HIDA scan shows a defect in the wall of gallbladder and extravasation of radioactive dye in peritoneal cavity [9].

Gallbladder perforation is usually managed with drainage of fluid, and cholecystectomy. Cholecystectomy can be performed after the infection is relieved by US-guided percutaneous drainage [9].

The mortality rate of gall bladder perforation was as high as 42% in a study by Glenn and Moore [11] while other studies by Derici et al. reported that morbidity and mortality rates were 37.5% and 12.5% from gall bladder perforation [9].

When in diagnostic dilemma, subtle clues such as free fluid in the abdomen and contracted gall bladder should raise suspicion of gall bladder perforation. Percutaneous cholecystostomy is usually practiced in unstable patient presenting with cholecystitis. However, in case of gall bladder perforation, percutaneous cholecystostomy alone may not be sufficient and drainage of intraperitoneal fluid pocket may act as a bridging therapy until definitive surgical management is done. It is vital to optimize the hemodynamics to prevent the downward spiral of end organ dysfunction until the source control is addressed. Every effort should be made to drain the fluid as even a minor intervention such as peritoneal fluid drainage with pigtail placement can reverse the trajectory of SOFA worsening as in our case [12]. The gall bladder was found to be necrotic later. The initial sepsis-like presentation was likely biliary peritonitis causing systemic inflammatory response syndrome (SIRS).

## Conclusions

GBP is a rare complication of cholecystitis with high morbidity and mortality. However, it is often difficult to diagnose when imaging modalities such as CT scan, ultrasonogram and HIDA scan are inconclusive and patients present late with sepsis and hemodynamic stability. In such cases, subtle clues such as free fluid in the abdomen with contracted gall bladder should be enough to raise suspicion of gall bladder perforation. Timely recognition and prompt intervention, as minor as peritoneal drainage, can result in unremarkable recovery and reduction in mortality.
